# Genomic studies in fragile X premutation carriers

**DOI:** 10.1186/1866-1955-6-27

**Published:** 2014-07-30

**Authors:** Reymundo Lozano, Randi J Hagerman, Michael Duyzend, Dejan B Budimirovic, Evan E Eichler, Flora Tassone

**Affiliations:** 1 MIND Institute, UC Davis Medical Center, Sacramento, 2825 50th Street, California, CA 95817, USA; 2 Department of Pediatrics, UC Davis Medical Center, Sacramento, CA, USA; 3 Department of Genome Sciences, University of Washington School of Medicine, Seattle, WA, USA; 4 Kennedy Krieger Institute, Johns Hopkins Medical Institutions, Baltimore, MD, USA; 5 Howard Hughes Medical Institute, Seattle, WA, USA; 6 Department of Biochemistry and Molecular Medicine, UC Davis Medical Center, Sacramento, CA, USA

**Keywords:** Premutation, *FMR1* gene, Autism, Second hit, ASD, Neurodevelopmental disorders, Neurological disorders

## Abstract

**Background:**

The *FMR1* premutation is defined as having 55 to 200 CGG repeats in the 5′ untranslated region of the fragile X mental retardation 1 gene (*FMR1*). The clinical involvement has been well characterized for fragile X-associated tremor/ataxia syndrome (FXTAS) and fragile X-associated primary ovarian insufficiency (FXPOI). The behavior/psychiatric and other neurological manifestations remain to be specified as well as the molecular mechanisms that will explain the phenotypic variability observed in individuals with the *FMR1* premutation.

**Methods:**

Here we describe a small pilot study of copy number variants (CNVs) in 56 participants with a premutation ranging from 55 to 192 repeats. The participants were divided into four different clinical groups for the analysis: those with behavioral problems but no autism spectrum disorder (ASD); those with ASD but without neurological problems; those with ASD and neurological problems including seizures; and those with neurological problems without ASD.

**Results:**

We found 12 rare CNVs (eight duplications and four deletions) in 11 cases (19.6%) that were not found in approximately 8,000 controls. Three of them were at 10q26 and two at Xp22.3, with small areas of overlap. The CNVs were more commonly identified in individuals with neurological involvement and ASD.

**Conclusions:**

The frequencies were not statistically significant across the groups. There were no significant differences in the psychometric and behavior scores among all groups. Further studies are necessary to determine the frequency of second genetic hits in individuals with the *FMR1* premutation; however, these preliminary results suggest that genomic studies can be useful in understanding the molecular etiology of clinical involvement in premutation carriers with ASD and neurological involvement.

## Background

As the *FMR1* premutation (55 to 200 CGG repeats) is common in the general population (1 in 130-259 females and 1 in 450-813 males) [1], the phenotypic manifestations of carriers may impact more than 1 million individuals in the US alone. Approximately 20% of female carriers have fragile X-associated primary ovarian insufficiency (FXPOI) [2], and 40% of male carriers and 8 to 16% of female carriers have fragile X-associated tremor/ataxia syndrome (FXTAS) [3,4].

In general, developmental problems in childhood occur in approximately 15 to 20% of premutation carriers. Premutation carriers identified through cascade testing following the diagnosis of a fragile X disorder in a proband showed that 8% have a diagnosis of autism spectrum disorder (ASD) and 30% of attention deficit hyperactivity disorder (ADHD) [5]. Approximately 70% of boys with the premutation who present clinically to a center with autism diagnostic testing have ASD, whereas 60% have ADHD and 20% have intellectual disability (ID) [5].

There are many reasons for the variability of clinical involvement in carriers. As the number of CGG repeats increases, the level of the encoded product of the *FMR1* gene (fragile X mental retardation protein; FMRP) decreases [6,7]. The low levels of FMRP are likely associated with both lower IQ and more emotional and behavioral problems [8,9]. In addition, the level of *FMR1* mRNA increases as the CGG repeat number increases [10] leading to RNA toxicity involving sequestration of important proteins for neuronal function, such as Sam 68, DROSHA and DGCR8 [11] The subsequent cascade of molecular events include upregulation of heat shock proteins [12]), dysregulation of Lamin A/C [13], deterioration of mitochondrial function [14,15] and the formation of potential toxic polypeptides [16]. Neuronal cell cultures of the premutation CGG mouse (knock-in; KI) showed altered dendritic branching, early death [12], enhanced spikes [17] and mitochondrial dysfunction [18]. Cunningham and collaborators [19] have also demonstrated abnormalities in neuronal migration during development in the premutation CGG mouse. This led us to hypothesize that patients with the *FMR1* premutation may be particularly susceptible to an *FMR1*-based ‘double hit’, which in addition to a second genetic hit will cause exacerbation of the clinical phenotype in carriers.

Copy number variants (CNVs), one of the sources leading to genetic variability in humans, can be responsible for Mendelian or sporadic traits but can also be associated with complex disorders. Indeed several studies have suggested that rare, large events can significantly contribute to the risk for a number of human disorders including ASD and ID [20-22]. Thus, we have investigated the role of genomic changes by assessing CNVs in premutation carriers to better understand the relationship with the observed clinical variability.

## Methods

### Study subjects

**Figure 1:**
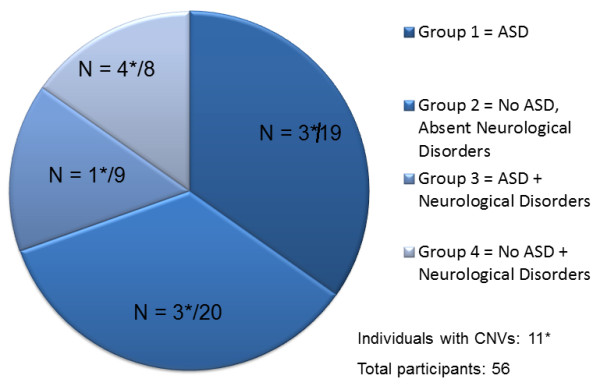
**Diagram of the distribution of the CNVs among participants in the four groups.** ASD, autism spectrum disorder; CNV, copy number variant.

Subjects with a premutation in *FMR1* were recruited through the Fragile X Treatment and Research Center at the UC Davis MIND Institute (Sacramento, CA, USA) according to a UC Davis Institutional Review Board (IRB) approved protocol and all signed consent for this study. All statistical analyses were completed using SPSS Statistics Version 21 (IBM Corporation, Armonk, NY, USA). Comparisons between groups were conducted using *t*-tests and chi-square tests, with a *P* value of less than 0.05 considered significant. A total of 56 patients with the premutation were recruited, four were females and 52 were males, and the mean age was 17.7 years old (SD 13.2 years). Of the total 56 patients: 19 had ASD (Group 1); 20 had neither ASD nor neurological problems but may have had ADHD, anxiety or other behavioral problems (Group 2); nine had ASD and neurological problems (Group 3); and eight had only neurological problems (Group 4) (Figure 1). The diagnosis of ASD was given according to the Diagnostic and Statistical Manual of Mental Disorders, 4th edition (DSM-IV) [23] and International Statistical Classification of Diseases and Related Health Problems, 10th revision (ICD-10) [24] criteria. Neurological problems included seizures, autonomic dysfunction, tremors, ataxia, weakness or paralysis. While most of the symptomatic participants were probands, most of the participants who did not have a diagnosis of ASD or neurological problems were non-probands, and only a few of these individuals were probands due to behavior problems (anxiety, ADHD and depression).

### Molecular measures

#### ***CGG sizing***

Genomic DNA was extracted from 3 to 5 ml of blood using standard procedure (Qiagen, Valencia, CA, USA). CGG repeat sizing was performed by PCR and Southern blot analysis as previously described [25,26].

#### ***Copy number variants (CNVs)***

Rare CNVs are defined as deletions and duplications occurring at a frequency <0.1% in the general population (8,328 controls). We utilized the genomic architecture of the human genome to identify regions of rare, recurrent CNVs. Segmental duplications are large blocks of sequences (>10 Kb) with high sequence identity (>95%) and interspersed in the human genome [27,28]. Segmental duplications, due to their high sequence identity, can therefore form substrates for unequal crossover during meiosis resulting in deletions or duplications of the intervening region, termed genomic hotspots [27,29]. We utilized a previously designed custom 12-plex NimbleGen array with a total of 135,000 probes targeted to genomic hotspots for CNV detection [30]. The hotspot array consists of a high density of probes (approximately 2.6 Kb) targeting 107 genomic hotspot regions (approximately 251 Kb) and a probe spacing of approximately 36 Kb in the genomic backbone. Array hybridization experiments and analysis were performed as described previously [21]. All signal intensities from the CGH experiments were loaded onto a UCSC Genome Browser mirror (Santa Cruz, CA, USA) and manually visualized. We also called CNVs using a hidden Markov model (HMM)-based segmentation algorithm from the normalized signal intensity data. This algorithm generates a normal distribution based on the probe signal intensities for each chromosome and generates z-scores (based on a 2 SD Gaussian model) for sets of probes that are either deleted or duplicated within that chromosome [30]. CNV calls were refined by applying the following filters: z-score < |1.5|, probes <10, >50% overlap with segmental duplications and length <50 Kb. We observed how many events had >50% reciprocal overlap with 8,328 controls [31].

### Cognitive and behavioral measures

The Autism Diagnostic Observation Schedule (ADOS) [32] and Autism Diagnostic Interview-Revised (ADI-R) [33] were used to diagnose ASD. Behavioral scales, the Vineland Adaptive Behavior Scales, 2nd edition (VABS-II) [34], and the Swanson, Nolan and Pelham, version IV (SNAP-IV) scale [35] for ADHD were also administered. The neuropsychological/neuropsychiatric assessments included standardized IQ tests, including different assessment methods: Stanford-Binet Intelligence Scales, 5th edition (SB-5) [36]; and Wechsler Adult Intelligence Scales (WAIS-III or WAIS-IV) [37,38].

## Results

A premutation was confirmed in all individuals and the CGG repeat size ranged from 55 to 192 repeats. Of the 56 subjects included in this study, 11 (19.6%) were found to have rare CNVs (average size = 508 Kb; Table 1): three of these were in Group 1 (ASD and no neurological disorders); three in Group 2 (no ASD and no neurological disorders); one in Group 3 (ASD with neurological disorders); and four in Group 4 (no ASD with neurological disorders) (Figure 1). The frequency of CNVs was not significantly higher in carriers with neurological signs compared to the premutation carriers without neurological disorders (5/17, 29.4% versus 6/39, 15.4%, χ^2^, df 1, *P* = 0.196) or among individuals with neurological disorders with and without ASD (1/9, 11.1% versus 4/8, 50%, χ^2^, df 1, *P* = 0.570); however, a trend was observed towards participants with neurological signs. From the 29 individuals with ASD, regardless of the presence of neurological disorders, four had a CNV (7.1%); and from the 27 individuals without ASD, seven had a CNV (12.5%, χ^2^, df 1, *P* = 0.211). A McNemar test (binominal distribution) showed a significant difference between the percentage of CNVs in neurologically affected individuals with and without ASD (n = 56, *P* = 0.035).

**Table 1:** Demographic, clinical and molecular measures

**Case**	**Gender**	**Age (years)**	**CGG repeat size**	***FMR1 *** **mRNA**	**Region**	**Start**	**End**	**Size**	**Inheritance**	**Controls n = 8,328 (50% reciprocal)**	**Gene(s)**	**Group**^**a**^	**Other clinical information**
1	M	12	64	2.19 (0.3)	Del 11q13.3	70,077,400	70,614,279	536 Kb	Unknown	0	*SHANK2*	1	Significant cognitive deficits, anxiety, tactile sensitivity, hyperactivity and perseveration
2	M	15	58	1.57 (0.06)	Del 6q26	162,565,963	162,748,669	182 Kb	Familial/ maternal	0	*PARK2*	1	Anxiety, hyperactivity, ID, tactile defensiveness, perseveration and aggression
3	M	5	81	2.41 (0.16)	Del 2q21.3	135,591,912	135,966,218	374 Kb	Familial/paternal	0	*RAB3GAP1*, *ZRANB3*	3	Severe autism, ADHD, obsessive behavior and EEG with generalized polyspikes during sleep
					Dup Xp22.31	6,442,757	8,115,638	1.67 Mb	Unknown	0	*VCX3A*, *HDHD1*, *STS*, *VCX*, *PNPLA4*, *MIR651*, *VCX2*		
4	M	9	66	1.95 (0.23)	Dup Xp22.31	6,404,592	6,907,093	502 Kb	Unknown	0	*VCX3A*	2	Anxiety, learning problems, trichotillomania, phobias and psychosis
5	F	69	20, 80	2.11 (0.2)	Dup 10q26.3	134,492,316	134,941,539	449 Kb	Not inherited to daughter	0	*C10orf93*, *GPR123*, *KNDC1*, *UTF1*, *VENTX*, *MIR202*, *ADAM8*	4	Severe autonomic dysfunction, tremor, ataxia and memory problems
6	F	35	29, 106	3.32 (0.44)	Dup 10q26.3	134,543,728	134,955,025	411 Kb	Not inherited to son	0	*C10orf93*, *GPR123*, *KNDC1*, *UTF1*, *VENTX*, *MIR202*, *ADAM8*, *TUBGCP2*	4	Severe autonomic dysfunction, ataxia, tremor, orthostatic hypertension and migraines
7	M	14	192	5.02 (0.49)	Dup 6p22.3	17,977,030	18,137,572	160 Kb	Unknown	0	*KIF13A*	1	Significant sensory integration issues, suicidal threats, hearing voices and frequent tantrums
8	M	7	99	2.98 (0.22)	Dup 10q26.2	128,750,641	129,507,857	757 Kb	Unknown	0	*DOCK1*, *FAM196A*, *NPS*, *FOXI2*	4	OCD and severe autonomic function
9	M	6	80	2.14 (0.1)	Dup 13q12.12	24,060,479	24,236,403	175 Kb	Familial/ maternal	0	*LOC374491*, *ATP12A*, *RNF17*	2	Severe behavior problems and tantrums
10	M	6	56	1.77 (0.1)	Del 21q21.2	23,370,733	23,780,822	410 Kb	Unknown	0	None	2	Anxiety, obsessive compulsive, tactile aversion and hyperarousal
11	F	31	29, 102	2.91 (0.03)	Dup 3q27.1	184,311,092	184,784,473	473 Kb	Unknown	0	*LAMP3*, *MCF2L2*, *B3GNT5*, *KLHL6*	4	Depression

^**a**^Group 1, ASD and no neurological disorders; Group 2, no ASD and no neurological disorders; Group 3, ASD with neurological disorders; and Group 4, no ASD with neurological disorders. *ADHD*, attention deficit hyperactivity disorder; *ASD*, autism spectrum disorder; *EEG*, electroencephalography; *F*, female; *M*, male; *OCD*, obsessive compulsive disorder.

The CNVs were eight duplications and four deletions, ranging from 175 Kb to 1.6 Mb; one individual had both a duplication (1.6 Mb) and a deletion (347 Kb). Five duplications ranging in size from 160 Kb to 1.6 Mb were found in patients with neurological problems. Interestingly, three of them were at 10q26, two of them overlapping (coordinates: 134,543,728 to 134,941,539) with duplication of genes *C10orf933*, G-protein-coupled receptor 123 (*GPR123*), *KNDC1*, undifferentiated embryonic cell transcription factor 1 (*UTF1*), Vent homeobox (*VENTX*), microRNA 202 (*MIR202*) and A disintegrin and metalloproteinase 8 (*ADAM8*). In addition, two individuals carried a duplication on Xp22.3 with a small area of overlapping (6,442,757 to 6,907,093), which included the *VCX3* gene. Only five of the 11 individuals had follow-up studies to determine if the CNVs were familial or *de novo*; three of them were followed by parental studies and all three were found to be inherited from asymptomatic parents (two maternal and one paternal); in the other two participants, parents were not available. We followed their offspring and the CNVs were not inherited to their asymptomatic children.

Taking in account all events (rare and common) after accurate filtering, total CNV burden analysis showed a significant enrichment of events >325 Kb was observed in premutation cases compared to controls (*P* = 2.274e-07).

Analysis of the psychometric assessments of all participants showed a mean full scale (FS) IQ of 83.20 (SD 23.0916) and ADOS total score of 4.45 (SD 6.53). Individuals with the premutation and a CNV had an ADOS mean score of 7.1 (SD 3.93) and FS IQ of 88.14 (SD 20.96). The participants without a CNV had an ADOS mean of 8.15 (SD 5.92) and FS IQ of 81.96 (SD 23.79), and these results were not statistically significant (ADOS, *P* = 0.6760 and IQ, *P* = 0.7218). Social Communication Questionnaire (SCQ) total score among individuals with a CNV had a mean of 13.88 (SD 9.5235) and among the individuals without a CNV had a mean of 11.5 (SD 9.8290), with no significant difference (*P* = 0.6398).

ADHD was found in 28/56 (50%) of individuals, of whom five had a CNV compared to 23/28 without a CNV. There were no significant differences in the CGG allele size between the group without a CNV (mean 90.95, SD 38.93) compared to those with a CNV (mean 84.85, SD 47.64, *P* = 0.4302).

We briefly describe six patients with genomic changes in more detail.

### Case 1

The patient was an adopted 12-year-old male with a premutation allele of 64 CGG repeats whose biological parents were not available. The patient had a diagnosis of ASD and severe behavior problems (Group 2). IQ was not available. CNV analysis showed the presence of a 536 Kb deletion in 11q13.3 involving the *SHANK2* gene, which encodes for a multi-domain molecular scaffolding protein enriched in neuronal synapses. *SHANK2* deletions have been associated with autism [39]. Moreover, it has been recently reported that the *SHANK2* mutant mouse recapitulates many of the behavioral phenotypes that are typical of ASD [40]. In this patient, the additional effects of the *SHANK2* deletion and the premutation may have caused ASD, but it is also a possibility that the deletion alone was responsible for the ASD.

### Case 2

The patient was a 15-year-old male with premutation of 58 CGG repeats. The patient had a diagnosis of anxiety, autism, ID (IQ = 54) and ADHD**.** The patient’s problem behaviors included frequent tantrums associated with aggressive episodes and hand flapping when excited or anxious. The patient’s physical examination was remarkable for broad knuckles, long tapered fingers and increased muscular tone. The patient was found to have a maternally inherited 180 Kb deletion in 6q26 that disrupted the *PARK2* gene. CNVs including the *PARK2* gene region have previously been reported in autism [41,42]. The *PARK2* gene encodes for the E3 ubiquitin-protein ligase, parkin, widely expressed in neuronal cells [43]. Parkin targets proteins for degradation in the cell. UBE3A, a protein from the same family, is associated with both autism and Angelman syndrome. *PARK2* has also been associated with mitochondrial function, particularly in protecting mitochondrial genomic integrity from oxidative stress [44]. Mitochondrial function is altered in subjects with autism [45], supporting parkin’s potential role in the pathophysiology of autism. The *PARK2* gene mutation is likely to have added to the baseline mitochondrial dysfunction in the premutation leading to ASD and ID. However, as *PARK2* variants have also been observed in individuals for the general population, the assessment of their pathogenicity can be quite complex.

### Case 3 and 4

These two patients were both males of 5 and 9 years of age (Table 1). Patient 3 had a premutation of 81 CGG repeats, duplication on Xp22.3 and a deletion on 2q21.3. This patient was diagnosed with autism, seizures and severe behavior problems. Case 4 had a premutation of 66 CGG repeats and duplication on Xp22.3. This patient was diagnosed with severe behavior problems but not with ASD. The duplication observed in these two cases had only one duplicated gene in common (*VCX3A*). The parents were not available for parental studies. The deletion of the *VCX3A* gene was initially reported to be associated with ID [46], but was found to be not sufficient to result in ID [47]. The duplication of these genes in addition to the premutation may have caused the more severe observed behavior problems.

### Case 5 and 6

Cases 5 and 6 were premutation carrier females (alleles with 80 and 106 CGG repeats, respectively, (Table 1), and were found to have overlapping duplicated regions of 449Kb and 411 Kb, respectively, at 10q26.3. They had similar clinical presentation including seizures, tremor, ataxia and autonomic dysfunction, which are common features of the FXTAS phenotype. While deletions of 10q26 have been associated with autism [48], the duplication on 10q26 has not been described to be pathogenic nor a benign CNV and was observed in only three cases of our CNV controls (3/8,328). It is also intriguing that this duplication was found in two individuals (Group 4) in this small cohort presenting with similar neurological phenotypes. These duplications were not inherited by their offspring. Little is known about the genes in the duplicated region; thus, further studies are necessary and may provide relevant information on these genes whose function may be relevant to neurodegenerative disorders including FXTAS.

## Discussion

**Figure 2:**
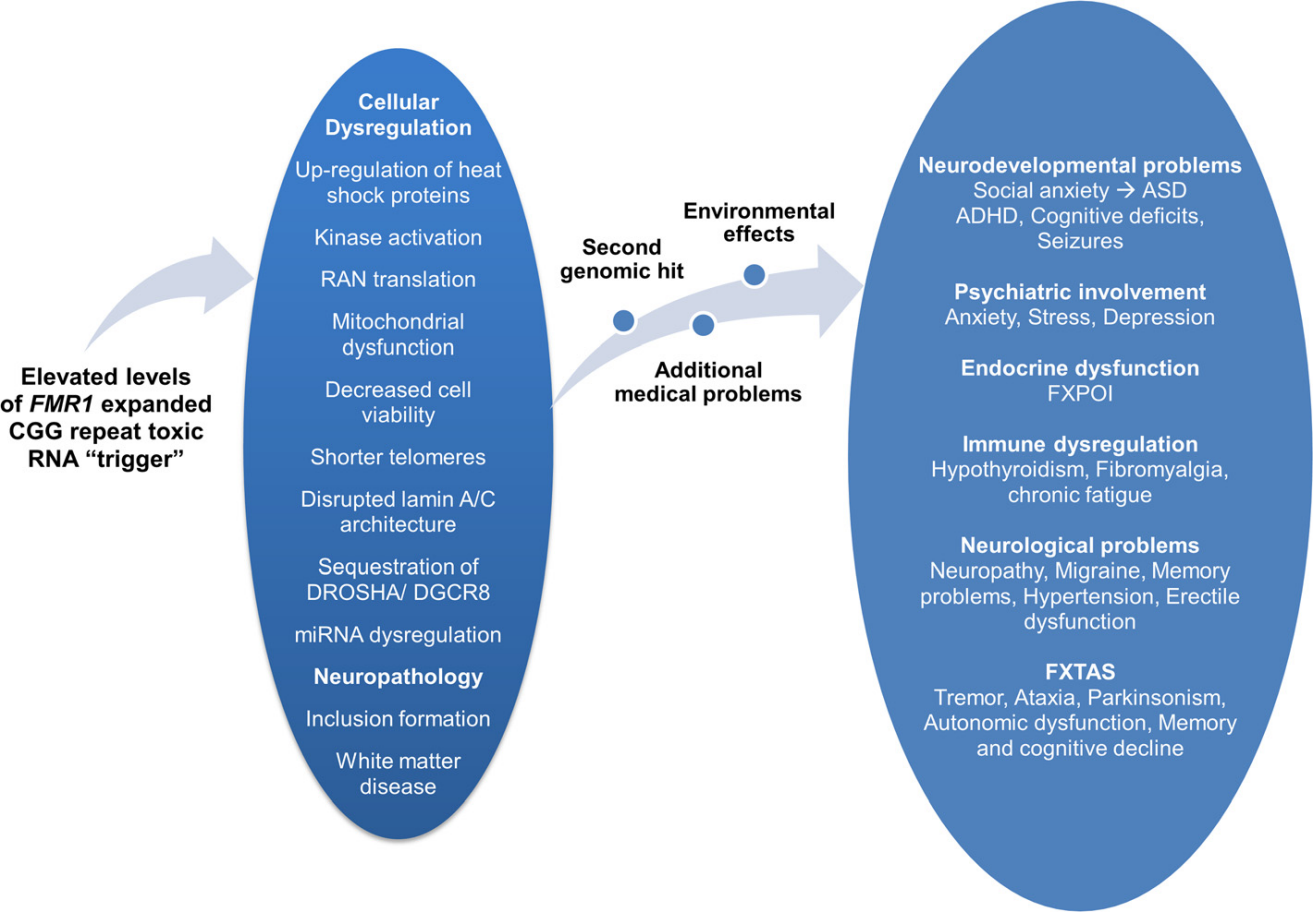
**Diagram of the clinical involvement in premutation carriers and potential players.** The *FMR1* premutation may represent a first hit that in addition to additional second hits may result in the variability of the phenotype. ADHD, attention deficit hyperactivity disorder; ASD, autism spectrum disorder; FXPOI, fragile X-associated primary ovarian insufficiency; FXTAS, fragile X-associated tremor/ataxia syndrome.

CNVs detected in recent studies of individuals with ASD have been shown to disrupt a number of genes that are collectively the cause of phenotypic variations [49]. The requirement of multiple genes for disease expression or multiple domains of expression of a largely monogenic disorder are manifestations of incomplete penetrance of any single gene; thus, mutations in multiple genes are required for full penetrance and for a more severe clinical phenotype [50]. Mutations within the *FMR1* gene can present with a variety of clinical phenotypes. For example, the premutation presents with the well characterized neurodegenerative disorder, FXTAS, and with FXPOI; however, it is also associated with other medical conditions. Some of these abnormalities are believed to be associated with RNA toxicity [51]. Remarkably, the neurological and neurodevelopmental disorders associated with the premutation have incomplete penetrance and variable expression even among families. This phenotypic variability suggests the participation of other ‘background’ modifier genes that when disrupted will cause additive effects.In addition, since the FMRP regulates the function of several genes, the premutation in combination with other genetic hits can cause other neurological and neurodegenerative disorders (Figure 2).

Along with the rapid genomic technological advances in the past few years, conceptual and technological challenges have emerged. It is important to clarify molecular techniques and their limitations, particularly in the clinical realm.

Microarrays and whole exome/genome sequencing cannot be compared as they involve different molecular techniques and are meant to detect different genetic abnormalities (CNVs versus single base pair changes). A second hit can also include a single base change, for example, in one of the studied participants, a non-verbal 22-year-old male with a premutation of 68 CGG repeats presented with autism, severe intellectual disability, seizures, macrocephaly and other mild facial dysmorphic features. While our CNV analysis did not detect the presence of a deletion or duplication, whole exome sequencing showed a *de novo* variant of uncertain clinical significance (c.4010_4034del21) in the *PTCH1* (patched *Drosophila* homolog) gene, which encodes for a 18 kDa histone H1-like protein, a transmembrane protein containing a patched-related domain with 12 transmembrane helices. A mutation within this gene has been observed in a child with ASD and Gorlin syndrome [52]; therefore, in this case the premutation and the *de novo* variant could have led to a more severe phenotype.

## Conclusion

To our knowledge this is the first study that shows rare CNVs in carriers of the *FMR1* premutation. A significant increase in the number of CNVs, specifically duplications, even after removal of rare and perhaps pathogenic events, has been found to be associated with autism [53]. The frequency of CNVs observed in the premutation with ASD is comparable to the rates seen in autism alone. Follow-up studies with an increased sample size are necessary to confirm and clarify these findings. Although preliminary, our overall results show the significant yield of genomic changes in individuals with the premutation presenting with neurological/neurodevelopmental disorders, including ASD. Future studies, including those that integrate a holistic molecular understanding of the interplay and consequences of *FMR1* genotype, mRNA and FMRP levels, in the context of detailed patient phenotypes, should further advance our understanding of the mechanism(s) underlying variable expression in premutation carriers.

### Consent

Subjects were recruited through the Fragile X Treatment and Research Center at the UC Davis MIND Institute (Sacramento, CA, USA) according to a UC Davis Institutional Review Board (IRB) approved protocol and all participants signed a consent for this study. Cases reported in the manuscript were consented for the report to be published.

## Abbreviations

*FMR1*: Fragile X mental retardation 1 gene; *ADAM8*: A disintegrin and metalloproteinase 8 gene; ADHD: Attention deficit hyperactivity disorder; ADI-R: Autism Diagnostic Interview-Revised; ADOS: Autism Diagnostic Observation Schedule; ASD: Autism spectrum disorder; CNV: Copy number variant; DSM-IV: Diagnostic and Statistical Manual of Mental Disorders, 4th edition; FMRP: Fragile X mental retardation protein; FS: Full scale; FXPOI: Fragile X-associated primary ovarian insufficiency; FXTAS: Fragile X-associated tremor/ataxia syndrome; *GPR123*: G-protein-coupled receptor 123 gene; HMM: hidden Markov model; ICD-10: International Statistical Classification of Diseases and Related Health Problems, 10th revision; ID: Intellectual disability; IQ: Intelligence quotient; IRB: Institutional Review Board; KI: Knock-in; *MIR202*: microRNA 202 gene; PCR: Polymerase chain reaction; SB-5: Stanford-Binet Intelligence Scales, 5th edition; SCQ: Social Communication Questionnaire; SD: Standard deviation; SNAP-IV: Swanson, Nolan and Pelham, version IV; *UTF1*: Undifferentiated embryonic cell transcription factor 1 gene; VABS-II: Vineland Adaptive Behavior Scales, 2nd edition; WAIS: Wechsler Adult Intelligence Scale.

## Competing interests

RH has received funding for treatment trials in fragile X syndrome or autism from Novartis (Basel, Switzerland), Roche (Basel, Switzerland), Seaside Therapeutics (Cambridge, MA, USA), Curemark (Rye, NY, USA), Forest Pharmaceuticals (New York City, NY, USA) and the National Fragile X Foundation (Walnut Creek, CA, USA). RH is also on the fragile X advisory boards for Novartis and Genentech/Roche. DH received support from Novartis, Roche and Seaside Therapeutics for clinical trials in fragile X syndrome. FT received research support from Roche. The Kennedy Krieger Institute (Baltimore, MD, USA) has received funding for treatment trials in fragile X syndrome from Novartis. EEE is on the scientific advisory boards for Pacific Biosciences, Inc (Menlo Park, CA, USA), SynapDx Corporation (Southborough, MA, USA) and DNAnexus, Inc (Mountain View, CA, USA). RL and MD have no conflicts of interest to disclose.

## Authors’ contribution

RL carried out the analysis of all the clinical and molecular data and participated to the writing of the manuscript. He also participated in obtaining clinical data. RH collected all clinical data and participated in the interpretation of all results and the analysis as well as participated to the writing of the manuscript. MD carried out the molecular CNVs studies and their analysis and participated to the writing of the manuscript. DB provided clinical data and and participated to the writing of the manuscript. EE designed the study, carried out the molecular CNVs studies and their analysis and participated to the writing of the manuscript. FT designed the study, carried out the molecular studies and their analysis. She overlook all the data analysis and interpretation as well as participated to the writing of the manuscript. All authors read and approved the final manuscript.

## References

[B1] Tassone F Iong KP Tong TH Lo J Gane LW Berry-Kravis E Nguyen D Mu LY Laffin J Bailey DB Jr Hagerman RJ FMR1 CGG allele size and prevalence ascertained through newborn screening in the United States Genome Med 2012 4 100 23259642 10.1186/gm401 PMC4064316

[B2] Sullivan AK Marcus M Epstein MP Allen EG Anido AE Paquin JJ Yadav-Shah M Sherman SL Association of FMR1 repeat size with ovarian dysfunction Hum Reprod 2005 20 402 412 15608041 10.1093/humrep/deh635

[B3] Coffey SM Cook K Tartaglia N Tassone F Nguyen DV Pan R Bronsky HE Yuhas J Borodyanskaya M Grigsby J Doerflinger M Hagerman PJ Hagerman RJ Expanded clinical phenotype of women with the FMR1 premutation Am J Med Genet A 2008 146A 1009 1016 18348275 10.1002/ajmg.a.32060 PMC2888464

[B4] Jacquemont S Hagerman RJ Leehey MA Hall DA Levine RA Brunberg JA Zhang L Jardini T Gane LW Harris SW Herman K Grigsby J Greco CM Berry-Kravis E Tassone F Hagerman PJ Penetrance of the fragile X-associated tremor/ataxia syndrome in a premutation carrier population JAMA 2004 291 460 469 14747503 10.1001/jama.291.4.460

[B5] Farzin F Perry H Hessl D Loesch D Cohen J Bacalman S Gane L Tassone F Hagerman P Hagerman R Autism spectrum disorders and attention-deficit/hyperactivity disorder in boys with the fragile X premutation J Dev Behav Pediatr 2006 27 S137 S144 16685180 10.1097/00004703-200604002-00012

[B6] Peprah E He W Allen E Oliver T Boyne A Sherman SL Examination of FMR1 transcript and protein levels among 74 premutation carriers J Hum Genet 2010 55 66 68 19927162 10.1038/jhg.2009.121 PMC4122982

[B7] Primerano B Tassone F Hagerman RJ Hagerman PJ Amaldi F Bagni C Reduced FMR1 mRNA translation efficiency in fragile X patients with premutations RNA 2002 8 1482 1488 12515381 PMC1370354

[B8] Hessl D Wang JM Schneider A Koldewyn K Le L Iwahashi C Cheung K Tassone F Hagerman PJ Rivera SM Decreased fragile X mental retardation protein expression underlies amygdala dysfunction in carriers of the fragile X premutation Biol Psychiatry 2011 70 859 865 21783174 10.1016/j.biopsych.2011.05.033 PMC3191264

[B9] Tassone F Hagerman RJ Ikle DN Dyer PN Lampe M Willemsen R Oostra BA Taylor AK FMRP expression as a potential prognostic indicator in fragile X syndrome Am J Med Genet 1999 84 250 261 10331602

[B10] Tassone F Hagerman RJ Taylor AK Gane LW Godfrey TE Hagerman PJ Elevated levels of FMR1 mRNA in carrier males: a new mechanism of involvement in the fragile-X syndrome Am J Hum Genet 2000 66 6 15 10631132 10.1086/302720 PMC1288349

[B11] Sellier C Freyermuth F Tabet R Tran T He F Ruffenach F Alunni V Moine H Thibault C Page A Tassone F Willemsen R Disney MD Hagerman PJ Todd PK Charlet-Berguerand N Sequestration of DROSHA and DGCR8 by expanded CGG RNA repeats alters microRNA processing in fragile X-associated tremor/ataxia syndrome Cell Reprogram 2013 3 869 880 10.1016/j.celrep.2013.02.004 PMC3639429 23478018

[B12] Chen Y Tassone F Berman RF Hagerman PJ Hagerman RJ Willemsen R Pessah IN Murine hippocampal neurons expressing Fmr1 gene premutations show early developmental deficits and late degeneration Hum Mol Genet 2010 19 1 196 208 19846466 10.1093/hmg/ddp479 PMC2792156

[B13] Garcia-Arocena D Yang JE Brouwer JR Tassone F Iwahashi C Berry-Kravis EM Goetz CG Sumis AM Zhou L Nguyen DV Campos L Howell E Ludwig A Greco C Willemsen R Hagerman RJ Hagerman PJ Fibroblast phenotype in male carriers of FMR1 premutation alleles Hum Mol Genet 2010 19 299 312 19864489 10.1093/hmg/ddp497 PMC2796892

[B14] Napoli E Ross-Inta C Wong S Hung C Fujisawa Y Sakaguchi D Angelastro J Omanska-Klusek A Schoenfeld R Giulivi C Mitochondrial dysfunction in Pten haplo-insufficient mice with social deficits and repetitive behavior: interplay between Pten and p53 PLoS One 2012 7 e42504 22900024 10.1371/journal.pone.0042504 PMC3416855

[B15] Ross-Inta C Omanska-Klusek A Wong S Barrow C Garcia-Arocena D Iwahashi C Berry-Kravis E Hagerman RJ Hagerman PJ Giulivi C Evidence of mitochondrial dysfunction in fragile X-associated tremor/ataxia syndrome Biochem J 2010 429 545 552 20513237 10.1042/BJ20091960 PMC4011071

[B16] Todd PK Oh SY Krans A He F Sellier C Frazer M Renoux AJ Chen KC Scaglione KM Basrur V Elenitoba-Johnson K Vonsattel JP Louis ED Sutton MA Taylor JP Mills RE Charlet-Berguerand N Paulson HL CGG repeat-associated translation mediates neurodegeneration in fragile X tremor ataxia syndrome Neuron 2013 78 440 455 23602499 10.1016/j.neuron.2013.03.026 PMC3831531

[B17] Cao Z Hulsizer S Cui Y Pretto DL Kim KH Hagerman PJ Tassone F Pessah IN Enhanced asynchronous Ca (2+) oscillations associated with impaired glutamate transport in cortical astrocytes expressing Fmr1 gene premutation expansion J Biol Chem 2013 288 13831 13841 23553633 10.1074/jbc.M112.441055 PMC3650419

[B18] Kaplan ES Cao Z Hulsizer S Tassone F Berman RF Hagerman PJ Pessah IN Early mitochondrial abnormalities in hippocampal neurons cultured from Fmr1 pre-mutation mouse model J Neurochem 2012 123 613 621 22924671 10.1111/j.1471-4159.2012.07936.x PMC3564636

[B19] Cunningham CL Martinez Cerdeno V Navarro Porras E Prakash AN Angelastro JM Willemsen R Hagerman PJ Pessah IN Berman RF Noctor SC Premutation CGG-repeat expansion of the Fmr1 gene impairs mouse neocortical development Hum Mol Genet 2011 20 64 79 20935171 10.1093/hmg/ddq432 PMC3000676

[B20] Eichler EE Flint J Gibson G Kong A Leal SM Moore JH Nadeau JH Missing heritability and strategies for finding the underlying causes of complex disease Nat Rev Genet 2010 11 446 450 20479774 10.1038/nrg2809 PMC2942068

[B21] Girirajan S Brkanac Z Coe BP Baker C Vives L Vu TH Shafer N Bernier R Ferrero GB Silengo M Warren ST Moreno CS Fichera M Romano C Raskind WH Eichler EE Relative burden of large CNVs on a range of neurodevelopmental phenotypes PLoS Genet 2011 7 e1002334 22102821 10.1371/journal.pgen.1002334 PMC3213131

[B22] Itsara A Wu H Smith JD Nickerson DA Romieu I London SJ Eichler EE De novo rates and selection of large copy number variation Genome Res 2010 20 1469 1481 20841430 10.1101/gr.107680.110 PMC2963811

[B23] American Psychiatric Association Diagnostic and Statistical Manual of Mental Disorders Text revision (DSM-IV-TR) 2000 4 Arlington, VA: American Psychiatric Association

[B24] World Health Organization International Statistical Classification of Diseases and Related Health Problems10th revision (ICD-10) 2010 Geneva: World Health Organization

[B25] Filipovic-Sadic S Sah S Chen L Krosting J Sekinger E Zhang W Hagerman PJ Stenzel TT Hadd A Latham GJ Tassone F A novel FMR1 PCR method for the routine detection of low-abundance expanded alleles and full mutations in fragile X syndrome Clin Chem 2010 56 399 408 20056738 10.1373/clinchem.2009.136101 PMC4031651

[B26] Tassone F Pan R Amiri K Taylor AK Hagerman PJ A rapid polymerase chain reaction-based screening method for identification of all expanded alleles of the fragile X (FMR1) gene in newborn and high-risk populations J Mol Diagn 2008 10 43 49 18165273 10.2353/jmoldx.2008.070073 PMC2175542

[B27] Bailey JA Gu Z Clark RA Reinert K Samonte RV Schwartz S Adams MD Myers EW Li PW Eichler EE Recent segmental duplications in the human genome Science 2002 297 1003 1007 12169732 10.1126/science.1072047

[B28] Sharp AJ Hansen S Selzer RR Cheng Z Regan R Hurst JA Stewart H Price SM Blair E Hennekam RC Fitzpatrick CA Segraves R Richmond TA Guiver C Albertson DG Pinkel D Eis PS Schwartz S Knight SJ Eichler EE Discovery of previously unidentified genomic disorders from the duplication architecture of the human genome Nat Genet 2006 38 1038 1042 16906162 10.1038/ng1862

[B29] Mefford HC Eichler EE Duplication hotspots, rare genomic disorders, and common disease Curr Opin Genet Dev 2009 19 196 204 19477115 10.1016/j.gde.2009.04.003 PMC2746670

[B30] Cooper JD Zerr T Kidd JM Eichler EE Nickerson DA Systematic assessment of copy number variant detection via genome-wide SNP genotyping Nat Genet 2008 40 1199 1203 18776910 10.1038/ng.236 PMC2759751

[B31] Cooper JD Coe BP Girirajan S Stevens H Burren OS Wallace C Greissl C Ramos-Lopez E Hypponen E Dunger DB Spector TD Ouwehand WH Wang TJ Badenhoop K Eichler EE A copy number variation morbidity map of developmental delay Nat Genet 2011 14;43 9 838 846 10.1038/ng.909 PMC3171215 21841781

[B32] Lord C Rutter M DiLavore PC Risi S Autism Diagnostic Observation Schedule (ADOS) 2000 Torrance, CA: Western Psychological Services

[B33] Lord C Rutter M Le Couteur A Autism Diagnostic Interview-Revised: a revised version of a diagnostic interview for caregivers of individuals with possible pervasive developmental disorders J Autism Dev Disord 1994 24 659 685 7814313 10.1007/BF02172145

[B34] Sparrow SS Cicchetti DV Balla DA Vineland Adaptive Behavior Scales 2005 2 Circle Pines, MN: AGS Publishing

[B35] Swanson JM Kraemer HC Hinshaw SP Arnold LE Conners CK Abikoff HB Clevenger W Davies M Elliott GR Greenhill LL Hechtman L Hoza B Jensen PS March JS Newcorn JH Owens EB Pelham WE Schiller E Severe JB Simpson S Vitiello B Wells K Wigal T Wu M Clinical relevance of the primary findings of the MTA: success rates based on severity of ADHD and ODD symptoms at the end of treatment J Am Acad Child Adolesc Psychiatry 2001 40 168 179 11211365 10.1097/00004583-200102000-00011

[B36] Roid G Barram R Essentials of Stanford-Binet Intelligence Scales (SB5) Assessment. Essentials of Psychological Assessment 2004 Hoboken, NJ: John Wiley & Sons

[B37] Wechsler D Wechsler Adult Intelligence Scale 3rd Edition (WAIS-III) 1997 3 Harcourt Assessment: San Antonio, TX

[B38] Wechsler D Wechsler Adult Intelligence Scale 4th Edition (WAIS-IV) 2008 4 Harcourt Assessment: San Antonio, TX

[B39] Berkel S Marshall CR Weiss B Howe J Roeth R Moog U Endris V Roberts W Szatmari P Pinto D Bonin M Riess A Engels H Sprengel R Scherer SW Rappold GA Mutations in the SHANK2 synaptic scaffolding gene in autism spectrum disorder and mental retardation Nat Genet 2010 42 489 491 20473310 10.1038/ng.589

[B40] Won H Lee HR Gee HY Mah W Kim JI Lee J Ha S Chung C Jung ES Cho YS Park SG Lee JS Lee K Kim D Bae YC Kaang BK Lee MG Kim E Autistic-like social behaviour in Shank2-mutant mice improved by restoring NMDA receptor function Nature 2012 486 261 265 22699620 10.1038/nature11208

[B41] Glessner JT Wang K Cai G Korvatska O Kim CE Wood S Zhang H Estes A Brune CW Bradfield JP Imielinski M Frackelton EC Reichert J Crawford EL Munson J Sleiman PM Chiavacci R Annaiah K Thomas K Hou C Glaberson W Flory J Otieno F Garris M Soorya L Klei L Piven J Meyer KJ Anagnostou E Sakurai T Autism genome-wide copy number variation reveals ubiquitin and neuronal genes Nature 2009 459 569 573 19404257 10.1038/nature07953 PMC2925224

[B42] Scheuerle A Wilson K PARK2 copy number aberrations in two children presenting with autism spectrum disorder: further support of an association and possible evidence for a new microdeletion/microduplication syndrome Am J Med Genet B Neuropsychiatr Genet 2011 156B 413 420 21360662 10.1002/ajmg.b.31176

[B43] Huynh DP Scoles DR Ho TH Del Bigio MR Pulst SM Parkin is associated with actin filaments in neuronal and nonneural cells Ann Neurol 2000 48 737 744 11079537

[B44] Rothfuss O Fischer H Hasegawa T Maisel M Leitner P Miesel F Sharma M Bornemann A Berg D Gasser T Patenge N Parkin protects mitochondrial genome integrity and supports mitochondrial DNA repair Hum Mol Genet 2009 18 3832 3850 19617636 10.1093/hmg/ddp327

[B45] Giulivi C Zhang YF Omanska-Klusek A Ross-Inta C Wong S Hertz-Picciotto I Tassone F Pessah IN Mitochondrial dysfunction in autism JAMA 2010 304 2389 2396 21119085 10.1001/jama.2010.1706 PMC3915058

[B46] Fukami M Kirsch S Schiller S Richter A Benes V Franco B Muroya K Rao E Merker S Niesler B Ballabio A Ansorge W Ogata T Rappold GA A member of a gene family on Xp22.3, VCX-A, is deleted in patients with X-linked nonspecific mental retardation Am J Hum Genet 2000 67 563 573 10903929 10.1086/303047 PMC1287516

[B47] Cuevas-Covarrubias SA Gonzalez-Huerta LM Analysis of the VCX3A, VCX2 and VCX3B genes shows that VCX3A gene deletion is not sufficient to result in mental retardation in X-linked ichthyosis Br J Dermatol 2008 158 483 486 18076704 10.1111/j.1365-2133.2007.08373.x

[B48] Yatsenko SA Kruer MC Bader PI Corzo D Schuette J Keegan CE Nowakowska B Peacock S Cai WW Peiffer DA Gunderson KL Ou Z Chinault AC Cheung SW Identification of critical regions for clinical features of distal 10q deletion syndrome Clin Genet 2009 76 54 62 19558528 10.1111/j.1399-0004.2008.01115.x

[B49] Iossifov I Ronemus M Levy D Wang Z Hakker I Rosenbaum J Yamrom B Lee YH Narzisi G Leotta A Kendall J Grabowska E Ma B Marks S Rodgers L Stepansky A Troge J Andrews P Bekritsky M Pradhan K Ghiban E Kramer M Parla J Demeter R Fulton LL Fulton RS Magrini VJ Ye K Darnell JC Darnell RB De novo gene disruptions in children on the autistic spectrum Neuron 2012 74 285 299 22542183 10.1016/j.neuron.2012.04.009 PMC3619976

[B50] Girirajan S Eichler EE Phenotypic variability and genetic susceptibility to genomic disorders Hum Mol Genet 2010 19 R176 R187 20807775 10.1093/hmg/ddq366 PMC2953748

[B51] Hagerman R Hagerman P Advances in clinical and molecular understanding of the FMR1 premutation and fragile X-associated tremor/ataxia syndrome Lancet Neurol 2013 12 786 798 23867198 10.1016/S1474-4422(13)70125-X PMC3922535

[B52] Delbroek H Steyaert J Legius E An 8.9 year old girl with autism and Gorlin syndrome Eur J Paediatr Neurol 2011 15 268 270 21190878 10.1016/j.ejpn.2010.12.001

[B53] Girirajan S Johnson RL Tassone F Balciuniene J Katiyar N Fox K Baker C Srikanth A Yeoh KH Khoo SJ Nauth TB Hansen R Ritchie M Hertz-Picciotto I Eichler EE Pessah IN Selleck SB Global increases in both common and rare copy number load associated with autism Hum Mol Genet 2013 22 2870 2880 23535821 10.1093/hmg/ddt136 PMC3690969

